# Production and Characterization of Biodiesel Using Nonedible Castor Oil by Immobilized Lipase from *Bacillus aerius*

**DOI:** 10.1155/2015/281934

**Published:** 2015-03-22

**Authors:** Sunil Kumar Narwal, Nitin Kumar Saun, Priyanka Dogra, Ghanshyam Chauhan, Reena Gupta

**Affiliations:** ^1^Department of Biotechnology, Himachal Pradesh University, Summer Hill, Shimla 171005, India; ^2^Department of Chemistry, Himachal Pradesh University, Summer Hill, Shimla 171005, India

## Abstract

A novel thermotolerant lipase from *Bacillus aerius* was immobilized on inexpensive silica gel matrix. The immobilized lipase was used for the synthesis of biodiesel using castor oil as a substrate in a solvent free system at 55°C under shaking in a chemical reactor. Several crucial parameters affecting biodiesel yield such as incubation time, temperature, substrate molar ratio, and amount of lipase were optimized. Under the optimized conditions, the highest biodiesel yield was up to 78.13%. The characterization of synthesized biodiesel was done through FTIR spectroscopy, ^1^H NMR spectra, and gas chromatography.

## 1. Introduction

Sustainability is a key principle in natural resource management and it has become increasingly obvious that continued dependence on fossil fuel energy resources is unsustainable in the long run because of depleting resources and the contribution of these fuels to environmental and health problems [1]. Thus transesterification of vegetable oils can be used for the synthesis of fatty acid methyl esters with properties similar to petroleum-based diesel fuel which is renewable source of energy and this process is increasingly researched as a means of producing an environmentally acceptable alternative fuel [2]. The use of lipases as biocatalysts in the transesterification of triacylglycerides allows mild reaction conditions and easy recovery of glycerol, without need for further purification or chemical waste production [3]. Enzymatic production of biodiesel by methanolysis of triglycerides using lipase as the catalyst offers several advantages compared to chemical processes. The cost of lipase production is one of the main obstacles for industrial application of lipases. Immobilization of lipases decreases cost of production by their reusability, which is necessary to make them more attractive and potent for industrial applications such as thermostability, activity in nonaqueous media, to improve handling, recovery, and recycling of biocatalyst [4]. Different vegetable oils are used for the biodiesel production, including sunflower oil [2, 5], waste cooking oil [6], soybean oil, [7], pomace oil [8], and palm oil [9]. Competition between food and biofuel leads to the search for fat sources which are not used as food, such as nonedible oils and restaurant waste lipids [10]. The production of biodiesel on a large scale using edible and nonedible oils promotes plantation of crops resulting in recycling of CO_2_ and minimizing its impact on the greenhouse effect [1].

In the present work, microbial lipase from* Bacillus aerius* was immobilized by adsorption onto silica matrix. Immobilized enzyme was used for biodiesel production by transesterification of castor oil with methanol. The effects of enzyme concentration, incubation time, relative molar concentration of reactants, and reaction temperature on the rate of synthesis of biodiesel were separately evaluated. Synthesized biodiesel from the methanolysis of castor oil was characterized by FTIR and NMR spectroscopy to get the evidence for the formation of products. No such research has been found in the literature using this organism which consequently leads to its novelty.

## 2. Materials and Methods

### 2.1. Chemicals and Enzyme

Silica gel matrix 60–150, glutaraldehyde,* p*-nitrophenyl acetate (*p*-NPA),* p*-nitrophenyl benzoate (*p*-NPB),* p*-nitrophenyl formate (*p*-NPF), and* p*-nitrophenyl palmitate (*p*-NPP) were purchased from Lancaster Synthesis, England; Tris buffer and castor oil were purchased from HIMEDIA Laboratory Ltd., Mumbai, India; and methanol was from MERCK, Mumbai, India. All chemicals were of analytical grade and were used as received. The lipase producing bacteria were isolated from the water sample of a hot spring named Tattapani, Kullu, Himachal Pradesh. The thermophilic* Bacillus aerius* (identified at IMTECH, Chandigarh) was grown in the medium of the following composition: yeast extract (2 g/L), peptone (5.0 g/L), sodium chloride (5.0 g/L), beef extract (1.5 g/L), ammonium chloride (1.0 g/L), and cottonseed oil (10 mL/L) (emulsified with 0.5% Gum Acacia) at pH 8.5. The seed culture (7.5% inoculum) was transferred to 50 mL production medium (250 mL Erlenmeyer flask) for 48 h under shaking conditions at 110 rpm at 55°C. The culture broth was centrifuged at 10,000 rpm for 10 min at 4°C. The lipase activity was assayed both in the supernatant as well as in pellet for determining extracellular and intracellular enzyme activity, respectively. The enzyme produced by thermophilic* Bacillus aerius* was purified to 9-fold with 7.2% recovery by ammonium sulfate precipitation and DEAE-cellulose column chromatography. The enzyme was found to be a monomeric protein having a molecular weight of 33 kDa on SDS-PAGE.

### 2.2. Determination of Lipase Activity

The activity of free and silica-bound lipase was measured by a colorimetric method [11]. The reaction mixture contained 60 *μ*L of* p*-nitrophenol palmitate (*p*-NPP) stock solution (20 mM* p*-NPP prepared in isopropyl alcohol) and 40 *μ*L lipase and Tris buffer (0.1 M, pH 9.5) to make final volume of 3 mL. The reaction mixture was incubated at 55°C for 10 min in a water bath. Keeping the reaction mixture at −20°C for 10 min stopped the reaction. The absorbance of* p*-nitrophenol released was measured at A_410_. The enzyme activity was defined as the micromoles of* p*-nitrophenol released per minute by the hydrolysis of* p*-NPP by 1 mL of soluble enzyme or 1 mg of silica-bound enzyme (weight of matrix included) under standard assay conditions. The protein was assayed by a standard method [12].

### 2.3. Immobilization of Lipase onto Silica

The silica gel matrix (60–150 mesh) was washed with 0.1 M Tris buffer (pH 7.0) and then centrifuged at 10,000 rpm at 4°C for 10 min. The supernatant was discarded and pellet was washed 4-5 times with Tris buffer. The matrix was then kept at 4°C overnight in Tris buffer. Then 1–5% glutaraldehyde (cross linking agent) solution was added to the 4 g matrix and kept at 35°C under shaking conditions for different time periods. The matrix was washed 3-4 times with Tris buffer (pH 7.0) to remove unbound glutaraldehyde. 8 mL (1.91 U/mL) of purified lipase from* Bacillus aerius* was then incubated with the matrix for 1 h under shaking condition. The supernatant was discarded.

### 2.4. Methanolysis of Castor Oil and Analysis of Biodiesel

Methanol (1 M) and nonedible castor oil (1 M) were taken in a screw-capped glass vial. To this mixture, enzyme (*Bacillus aerius *lipase ~1.44 U/mg) as a catalyst was added and incubated with constant shaking at 250 rpm. The effects of reaction time (24–120 h), temperature (40 and 60°C), oil to methanol molar ratio (1 : 1 to 1 : 6), and immobilized lipase amount (1–7% of oil weight) on biodiesel production were investigated in the reaction system. The reaction mixture was washed with distilled water to remove the glycerol and excess methanol. The quantification of ester was done by FTIR spectroscopy on Nicollet 5700 in KBr pallets, ^1^H NMR was done (Advance Buker II-400 MHz) in deuterated chloroform (CDCl_3_) solution with internal standard TMS (0 ppm), and chemical shifts were recorded in parts per million (*δ*/ppm) and GLC equipped with a flame ionization detector and a column (10% SE-30 Chrom WHP, 2-meter length, mesh size 80–100, internal diameter 1/8 inches, and maximum temperature limit 300°C; Netel Chromatograph, Thane, India). Nitrogen was used as a carrier gas (30 cm^3^/min). The injector was warmed to 250°C, and the detector was set at 280°C. The quantification was accomplished by intern standardization. Methyl ricinoleate was the internal standard used. The methyl ester yield was determined using GLC and % yield method as follows:

(1) $$\begin{array}{c} \%\;\text{yield}=\frac{\text{weight}\;\text{of}\;\text{biodiesel}\;\text{formed}\;\left(\text{g}\right)\;}{\text{Total}\;\text{weight}\;\text{of}\;\text{reaction}\;\text{mixture}\;\left(\text{g}\right)}\times 100. \end{array}$$

## 3. Results and Discussion

In the present study lipase activity from* Bacillus aerius* was found to be 1.44 U/mg and this activity is comparable with that reported in the previous study [4].

### 3.1. Effect of Reaction Time on Transesterification

**Figure 1:**
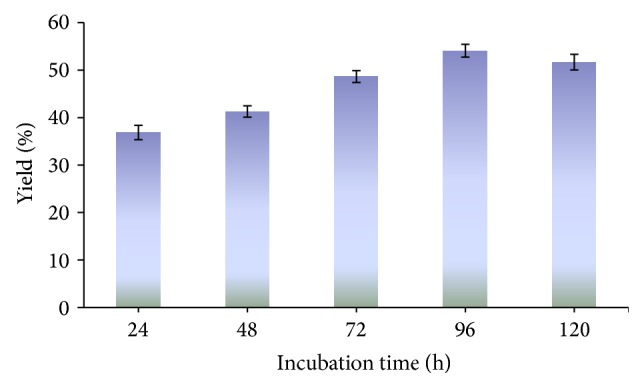
Time course of biodiesel synthesis tested at 55°C. Reaction conditions: methanol/oil 1 : 1 (mol/mol); 2% lipase by oil weight.

The effect of reaction time on the transesterification was investigated by varying the reaction time from 24 to 120 h. The yield of biodiesel increased on increasing incubation time from 24 to 96 h (Figure 1). At 96 h, approximately 54.08% of biodiesel was produced. Thus in the subsequent transesterification reactions, a reaction time of 96 h at 55°C for immobilized-lipase was considered optimum for the synthesis of biodiesel. Longer reaction time led to the reduction of biodiesel formation, because the transesterification reaction reverses and thus results in loss of methyl esters as well as soap formation [13, 14]. Li et al. observed the incubation time of 72 h to be optimal for the synthesis of biodiesel [15]. Since the activity of different lipases is different so Li et al. found 72 h as optimum incubation time as compared to 96 h.

### 3.2. Effect of Reaction Temperature

**Figure 2:**
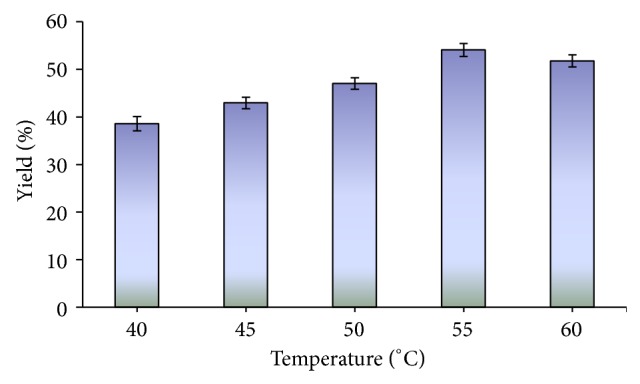
Effect of temperature on the synthesis of biodiesel. Reaction conditions: reaction time 96 h, methanol/oil 1 : 1 (mol/mol) and 2% lipase by oil weight.

Changes in the reaction temperature can affect the activity and stability of the enzyme and thus the rate of reaction. The effect of temperature on the lipase activity was examined using a temperature range of 40°C to 60°C as shown in Figure 2 with castor oil (1 M) and methanol (1 M) in a solvent free system for 96 h. The maximum biodiesel synthesis (54.08%) was observed at 55°C (Figure 2) under the above reaction conditions. Previously the optimum reaction temperature of 60°C was observed for the synthesis of biodiesel [16]. In a recent study, the optimum temperature of 37°C was observed for the synthesis of biodiesel [17], so this shows mesophilic nature of enzyme but the lipase from* Bacillus aerius* was thermotolerant; therefore maximum biodiesel production was observed at 55°C.

### 3.3. Effect of Methanol/Castor Oil Molar Ratio

**Figure 3:**
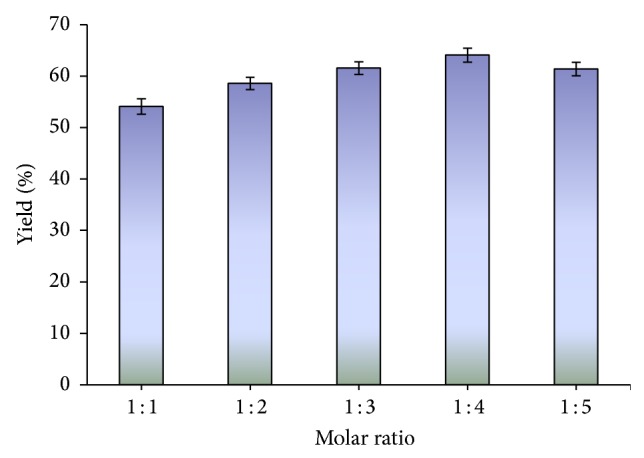
The effect of methanol/oil molar ratio on the methanolysis of castor oil. Reaction conditions: reaction temperature 55°C, 2% lipase by oil weight, and reaction time 96 h.

One of the most important variables affecting the yield of ester is the molar ratio of methanol to oil. A set of experiments was performed in which the oil/methanol molar ratio was varied in the range 1 : 1–1 : 5 (mol/mol). The results obtained (Figure 3) showed that the methyl ester yield (64.08%) was highest at 1 : 4 oil/methanol molar ratio. However further increase in molar ratio beyond the optimal level led to decrease in biodiesel yield. This might be due to the deactivation of the lipase by exposure to methanol. In a recent study, the 1 : 4 substrate molar ratio was found to be the best for the maximum yield of biodiesel [17]. Previously 6 : 20 (oil : methanol) molar ratio was found to be optimum for the synthesis of biodiesel [18].

### 3.4. Effect of Amount of Lipase

**Figure 4:**
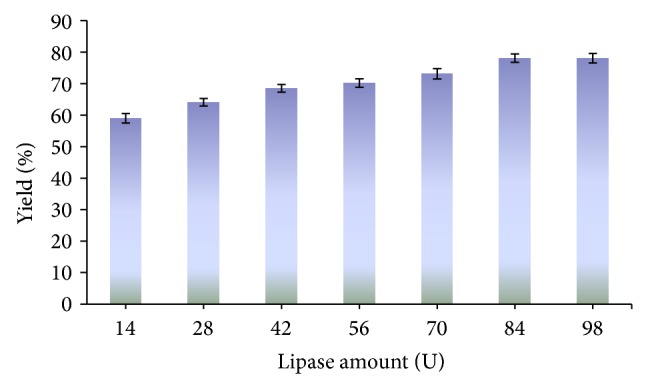
Effect of concentration of lipase on the synthesis of biodiesel. Reaction conditions: reaction time 96 h, reaction temperature 55°C, and methanol/oil 1 : 4 (mol/mol).

The influence of enzyme quantity on the methanolysis of castor oil has been shown in Figure 4. The maximum yield of biodiesel (78.13%) was obtained with 84 U of immobilized lipase. It was observed that, with an increase of enzyme concentration, initial rate of esterification was increased from 14 to 84 U and remained almost constant with increase in lipase amount beyond 84 U. The castor oil contains 85–90% of ricinoleic acid; thus methyl ricinoleate was used as internal standard.

### 3.5. Characterization of Ester by Analytical Methods

**Figure 5:**
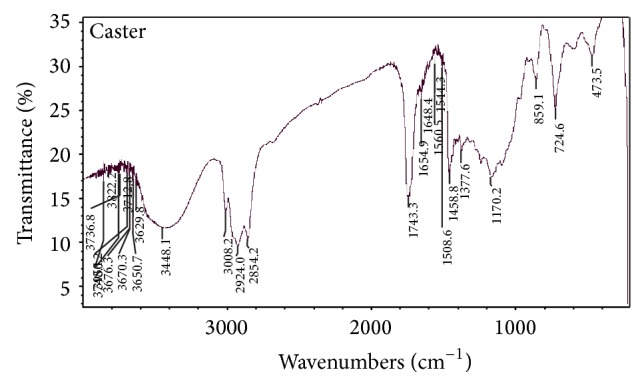
FTIR spectrum of transesterified castor oil.

**Figure 6:**
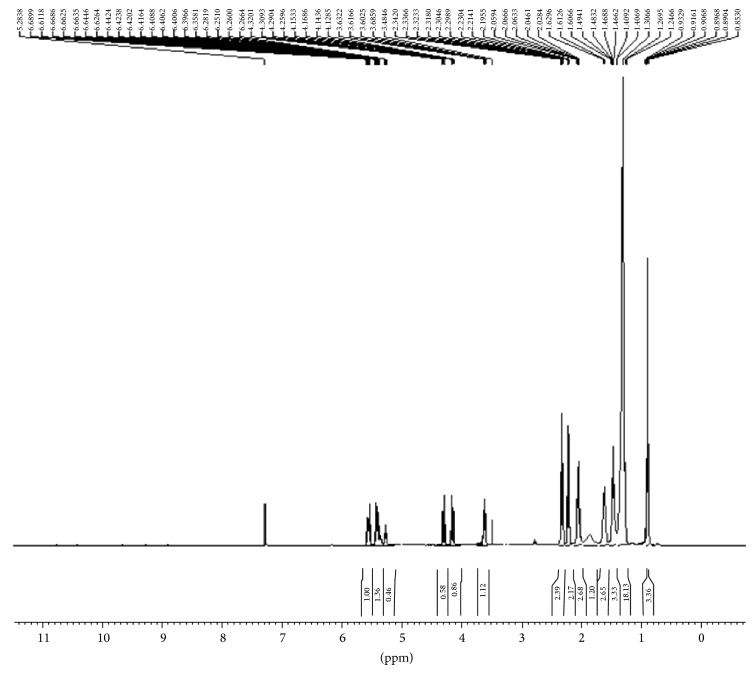
NMR spectrum of transesterified castor oil.

The Characterization of ester was also done by various analytical/spectroscopic methods which are FTIR, ^1^H NMR spectroscopy (Table 1) and gas chromatography. The FTIR spectrum of methyl ricinoleate shows the peak at 1743.39 cm^−1^ which is due to (–C=O stretching of ester), at 1648 cm^−1^ due to the (–C=C– stretching) but it has high intensity as the presence of ester group resulted in electronegativity of the neighboring groups and a peak at value 2942 cm^−1^ (–C–H– stretching) clearly confirms the formation of biodiesel (Figure 5). But when the above spectra were compared with their precursors in literature it was clearly found that the peak at value 1680 cm^−1^ is absent in Figure 5 which was due to –COOH group clearly confirming that esterification took place at this position and all other peaks remained; only the intensity of the peaks changed because of some new functional groups added in it. The ^1^H NMR spectra of methyl ester are shown in Figure 6. Signals at values 2.32 ppm and 4.21 ppm were due to presence of –CH_2_–O–C–O and –C=O functional groups of ester bond which were absent in the spectrum of their precursor. Also in the precursor molecule a signal at value 12.05 ppm is due to –OH group but in its ester form this signal is absent clearly confirming that the reaction took place at this position. All other peaks remained the same. The spectra of the products formed at various steps were matched with the ChemDraw Ultra 10 which unambiguously confirms the formation of biodiesel and also compared with its precursor molecules. From the % yield method and GLC analysis, it was observed that the yield of biodiesel produced was approximately the same.

## 4. Conclusion

Methanolysis of nonedible castor oil with lipase immobilized on silica to yield biodiesel through transesterification has been investigated. Biodiesel was successfully synthesized by silica-bound lipase in 96 h under shaking at 55°C using 1 : 4 oil/methanol molar ratio. Effect of the interaction among the different parameters for transesterification and enzyme kinetics was needed to be known to catalyze a reaction.* Bacillus aerius* lipase showed promising results and immobilizing the enzyme on inexpensive silica matrix could reduce cost as well as improve yield of ester.

## Figures and Tables

**Table 1:** Characteristic peaks of methyl ricinoleate taken as a standard.

S. number	Compound	FTIR peaks		NMR peaks	
		Functional groups	Values in cm^−1^	Functional groups	Values in ppm
1	Methyl ricinoleate	–C=O, OH	1743.39, 3481	–CH_2_–O–C–O, –C=O	2.32, 4.21
		–C=C–	1648	–C=C–	5.32
		–CH– stretching	2942	–CH_2_, –CH–	2.82, 1.29

2	Ricinoleic acid	–C=O, OH	1680, 3481	–C=O, OH	6.00, 12.05
		–C=C–	1616	–C=C–	5.42
		–CH–	2942	–CH_2_, –CH	2.52, 1.29
